# Five‐year outcomes of TOOKAD® (WST‐11) vascular‐targeted photodynamic therapy for low‐risk prostate cancer patients: Insights from a tertiary referral centre

**DOI:** 10.1002/bco2.70106

**Published:** 2025-12-17

**Authors:** Andrea Cosenza, Francesco Barletta, Leonardo Quarta, Michele Brancaccio, Giorgio Gandaglia, Francesco Montorsi, Alberto Briganti, Armando Stabile

**Affiliations:** ^1^ Unit of Urology/Division of Oncology, Gianfranco Soldera Prostate Cancer Lab IRCCS San Raffaele Scientific Institute Milan Italy; ^2^ Vita‐Salute San Raffaele University Milan Italy

Prostate cancer (PCa) is most commonly diagnosed in its localized stage, with radical treatments such as prostatectomy or radiotherapy offering high rates of cancer control.^1^ However, these curative options are frequently associated with significant adverse effects, including erectile dysfunction and urinary incontinence, which can negatively impact patients' quality of life.^2^ In response, interest in organ‐sparing approaches has increased, particularly for patients with low‐risk disease where overtreatment remains a pressing concern. Focal therapies (FTs) aim to treat only the cancerous portion of the prostate identified by targeted biopsy and multiparametric MRI, thereby reducing the burden of treatment‐related morbidity while maintaining oncological safety.

Among available FT modalities, vascular‐targeted photodynamic therapy (VTP) using TOOKAD® Soluble (WST‐11) has emerged as a promising option. VTP induces localized ablation of prostate tissue through the photochemical activation of a photosensitizer with near‐infrared laser light, resulting in vascular occlusion and subsequent tumour necrosis.^3^ While encouraging short‐term oncological outcomes have been reported, long‐term data remain limited.^4^

Here, we report 5‐year clinical outcomes of patients with low‐risk PCa treated with VTP at our tertiary referral centre.

Between 2018 and 2020, a total of 13 patients underwent VTP at San Raffaele Hospital. Eligibility criteria included unilateral ISUP Grade Group 1 disease on biopsy, PSA < 10 ng/mL and clinical stage cT1. All procedures were performed by a single experienced urologist. Data were prospectively collected and approved by the Institutional Review Board. Primary outcomes were technical feasibility, defined as the absence of Grades 4 and 5 adverse events, and PCa recurrence or progression necessitating radical treatment.

At treatment, median patient age was 65 years (IQR: 62–68), and median PSA was 6 ng/mL (IQR: 4–8). Multiparametric prostate MRI was available in 11 (85%) patients. Suspicious lesions were categorized as PI‐RADS 2–4 in 2 (18%), 3 (27%) and 6 (55%) patients, respectively, and showed concordance with biopsy‐identified cancer locations. Median prostate volume, measured via MRI or transrectal ultrasound, was 52 cc (IQR: 31–81). The median number of biopsy cores was 15 (IQR: 13–16), with three positive cores (IQR: 2–4). Time from biopsy to treatment was 6 months (IQR: 3–14).

All patients were discharged the day after the procedure. No serious (Grades 4 and 5) adverse events were recorded. One patient (7.7%) experienced acute urinary retention, while three (23%) had transient haematuria, all classified as Grade ≤3 events.

After a median follow‐up of 70 months (IQR: 52–72), five patients (38%) experienced recurrence. In Kaplan–Meier analysis, the 5‐year PCa recurrence‐free survival was 64% (95% CI: 41% to 99%), as reported in Figure 1. All recurrences were ISUP Grade Group 1 and occurred in field. Notably, only one patient underwent radical prostatectomy during follow‐up, resulting in a 5‐year treatment‐free survival rate of 90% (95% CI: 73% to 100%).

**FIGURE 1:**
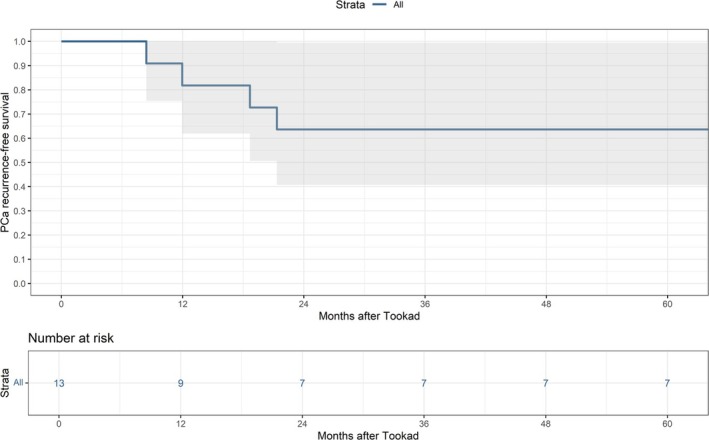
Kaplan–Meier plot depicting prostate cancer (PCa) recurrence‐free survival.

These findings support the oncological safety and feasibility of VTP in carefully selected patients with low‐risk PCa. The majority of men remained free from clinically relevant recurrence and avoided radical treatment over a 5‐year period. Importantly, no significant safety concerns emerged during extended follow‐up, underscoring the tolerability of the procedure.

Our findings align with prior evidence. Azzouzi et al.,^5^ in the CLIN1001 PCM301 phase III trial, reported a median time to recurrence of 28 months with a PCa‐free survival rate of 63%, similar to the rate reported in our cohort at a later time point. The slightly better long‐term outcomes observed in our study may reflect more rigorous initial patient selection and the integration of prostate MRI into the diagnostic work‐up, which enabled more accurate risk stratification. Indeed, 85% of our patients had MRI‐confirmed lesions concordant with biopsy results, potentially improving treatment targeting and reducing the risk of missed multifocal disease.

It is also worth noting that in the PCM301 trial, all patients underwent repeat biopsy or MRI within the first year. In our cohort, follow‐up imaging and biopsy decisions were individualized and performed based on clinical suspicion. While this may introduce a degree of ascertainment bias and possibly overestimate recurrence‐free survival, it reflects real‐world clinical practice, where overtreatment and over‐surveillance of low‐risk disease are increasingly questioned.

The 90% 5‐year treatment‐free survival in our cohort also compares favourably with rates observed in active surveillance (AS) cohorts. In the PCM301 trial, 29% of patients in the AS arm proceeded to radical treatment within 2 years, compared to only 6% in the VTP arm. Our results, consistent with these findings, reinforce the potential of VTP to delay or even avoid definitive therapy in men with indolent disease, without compromising safety.

Nonetheless, several limitations must be acknowledged. The small sample size and single‐centre design limit generalizability. The lack of standardized follow‐up protocols and absence of systematic post‐treatment biopsy data further constrain interpretation. In addition, we were unable to report functional outcomes such as urinary and sexual function, which are highly relevant in this context. Although the absence of serious complications is reassuring, structured functional assessment using validated instruments (e.g., IPSS and IIEF‐5) would strengthen future studies.

Despite these limitations, our study offers valuable insight into the long‐term safety and efficacy of VTP. To our knowledge, this is among the longest follow‐up series of low‐risk PCa patients treated with TOOKAD® Soluble VTP in a real‐world setting. The data highlight that a majority of patients remain recurrence‐free and avoid definitive therapy 5 years after treatment, suggesting durable disease control in this subset.

In this single‐centre series, VTP for low‐risk PCa showed favourable 5‐year outcomes, with 64% of patients free from PCa recurrence and 90% avoiding radical treatment. These results support VTP as a viable FT option for selected patients and contribute to the growing evidence base advocating for less invasive strategies in the management of low‐risk PCa.

## CONFLICT OF INTEREST STATEMENT

The authors declare no conflicts of interest in preparing this article.
